# DNAJA1 Stabilizes EF1A1 to Promote Cell Proliferation and Metastasis of Liver Cancer Mediated by miR-205-5p

**DOI:** 10.1155/2022/2292481

**Published:** 2022-05-09

**Authors:** Lizhi Yi, Shunhui He, Zhengyu Cheng, Xi Chen, Xiaoli Ren, Yang Bai

**Affiliations:** ^1^Guangdong Provincial Key Laboratory of Gastroenterology, Department of Gastroenterology, Institute of Gastroenterology of Guangdong Province, Nanfang Hospital, Southern Medical University, Guangzhou, China; ^2^Department of Gastroenterology, People's Hospital of Leshan, Leshan, 614000 Sichuan Province, China; ^3^Department of Gastroenterology, Shunde Hospital, Southern Medical University, Foshan, 528300 Guangdong Province, China; ^4^Department of Ultrasound, Sichuan Provincial Maternity and Child Health Care Hospital, Chengdu, 610045 Sichuan Province, China; ^5^Department of Pathology, The Affiliated Hospital of Southwest Medical University, Luzhou, 646000 Sichuan Province, China

## Abstract

Liver cancer is one of the most common and aggressive malignancies worldwide with poor prognosis. Studies on pathogenesis of liver cancer are urgently demanded to develop better treatment strategy. Here, we found that overexpression of DnaJ heat shock protein family (Hsp40) member A1 (DNAJA1) increased cell proliferation, invasion, and angiogenesis in Huh 7 and HepG2 cells, while depletion of DNAJA1 in MHCC-97H and HCC-M3 showed opposite effects. In vivo functional assays indicated that DNAJA1 promoted tumor growth and pulmonary metastasis in mice. Mechanistically, as a direct target of miR-205-5p, DNAJA1 promoted proliferation and metastasis of liver cancer cells by stabilizing eukaryotic elongation factor 1A1 (EF1A1). Moreover, DNAJA was markedly upregulated in liver cancer tissues (*P* < 0.05) and was significantly associated with poor prognosis. And its expression was correlated with differentiation (*P* < 0.001), dissemination (*P* < 0.001), and serum AFP (*P* = 0.029). The mRNA levels of miR-205-5p and DNAJA1 were negatively correlated in liver cancer. In conclusion, our study reveals that DNAJA1 acts as an oncogene in liver cancer via miR-205-5p/EF1A1 axis and might be a potential biomarker to predict the prognosis for liver cancer patients.

## 1. Introduction

Liver cancer is one of the most aggressive carcinomas globally [1, 2]. Although the prognosis of patients with liver cancer has been improved with the development of surgical techniques, the poor overall survival of patients with liver cancer still exists owing to the high rate of mortality [3–5]. Till now, a variety of molecules have been reported to contribute to the initiation and progression of liver cancer [6]. Therefore, it is of great significance to seek useful biomarkers, predict prognosis, and explore therapeutic strategies for liver cancer patients.

DNAJA1, a member of DNAJ/HSP40 family proteins, is involved in variety of cellular life activities, such as protein degradation, translocation, and folding [7, 8]. Studies have shown that DNAJA1 also participated in the tumor progression. It was upregulated in colorectal cancer and associated with poor prognosis and distant metastasis [9]. DNAJA1 could induce the radiotherapy resistance of glioblastomas [10] and anticancer drug resistance [11] and suppressed the stress response capabilities of c-Jun and cell survival in pancreatic cancer [12]. However, the role of DNAJA1 in liver cancer is still not well understood. Our study shows that DNAJA1 was upregulated in liver cancer tissues and associated closely with poor prognosis, suggesting a role as an oncogene in proliferation and metastasis of liver cancer cells.

## 2. Materials and Methods

### 2.1. Human Specimens and Cell Lines

106 cases of paraffin-embedded liver cancer specimens were collected from patients who accepted the operation of liver cancer resection without prior radiotherapy and chemotherapy at the Department of Hepatobiliary Surgery in Nanfang Hospital in 2014-2016 year. All patients were followed up for at least 5 years. 43 cases of fresh liver cancer tissues were collected immediately after resection, snap-frozen in liquid nitrogen, and then stored at -80°C.

Human liver cancer cell lines HCC-M3, HepG2, Hun7, MHCC97H, and Hep 3B were purchased from American Type Culture Collection (ATCC) and authenticated by STR profiling. All cell lines were cultured in Dulbecco's modified Eagle medium (DMEM) (GIBCO, Gaithersburg, MD, USA) with 10% fetal bovine serum (HyClone, Logan, USA) at 37°C with 5% CO_2_.

### 2.2. Subcutaneous Tumor Implantation and Metastasis Assays

4-6-week-old male athymic BALB/c-nu/nu mice were obtained from the Central Laboratory of Animal Science at Southern Medical University (Guangzhou, China). 1 × 10^5^ cells of HCC-M3 cells with or without DNAJA1 shRNA expression were injected into nude mice for subcutaneous tumor implantation, and mice were allowed to grow for 28 days before sacrifice. For metastasis assays, 5 × 10^4^ HCC-M3 cells with or without DNAJA1 shRNA were injected within the liver capsular for 40 days. All tissues were embedded in paraffin, sectioned, stained with hematoxylin and eosin, or subjected to immunohistochemistry (IHC) staining.

### 2.3. Plasmid Structure and Transfection

For depletion of DNAJA1, the lentivirus vectors carrying two human shRNAs toward DNAJA1 (GeneCopoeia, Guangzhou, China) was transfected into lentiviral packaging cell lines 293T cells. The shRNA sequences were as follows: shRNA1 (sense, 5′-GACCGAATTTCACTACAAA-3′) and shRNA2 (sense, 5′-GGTTGAACTTGCTAGTGAT-3′) (Invitrogen, Foster City, CA, USA). A scramble shRNA (5′-AATCGCATAGCGTATGCCGTT-3′) that has no homology with the mammalian mRNA sequences was inserted into empty lentivirus vector as described above. Then, 1 ml of viral supernatant containing 4 Ag of polybrene was added into liver cancer cell lines for stable transduction. After 14 days, puromycin-resistant cell pools were established. After 72 h, the protein level of DNAJA1 was detected by western blotting. For overexpression plasmid of DNAJA1, the fragment of human DNAJA1 was amplified from human cDNA by PCR (forward primer: 5′-AAAGGTACCATGAAGAATGAAATTGCTGCCGTTG-3′; reverse primer: 5′-ACTCGAGTT AGTGAGGTGCTAACATGTGAGGA-3′). The fragment was digested with Kpn1 and Xho1 and cloned into the Kpn1-Xho1 site of the pEGFP-C1 expression vector. Then, pEGFP-C1-DNAJA1 vector or its control vector was transfected into cells with Lipofectamine 3000.

### 2.4. Cell Proliferation Assay

1 × 10^3^ cells were seeded into 96-well plates.10 *μ* l CCK-8 solution was added into 90*μ* l DMEM with 10% fetal bovine serum after 12 hours followed by incubation at 37°C for 2 hours and absorbance measurement at 490 nm. The tests were continued for 6 days. For plate colony assay, 800 cells were seeded into 6-well plates and cultured at 5% CO_2_, 37°C for 2 weeks. The number of colonies (each colony > 50 cells) were counted and then stained with hematoxylin. Five duplicate wells were plated for each group.

### 2.5. In Vitro Invasion Assay

600 *μ*l RPMI 1640 containing 10% fetal bovine serum was added to the lower compartments of the rehydrated chamber, and then, 1.5 × 10^5^ tumor cells in serum-free DMEM were added to the upper compartment. After incubation for 48 hours, the noninvasive cells were wiped and cells that had migrated through the membrane were stained with hematoxylin and counted under a light microscope in 5 random visual fields (200x). Each experiment was repeated three times.

### 2.6. Tube Formation Assay

Matrigel matrix (Corning) was plated onto angiogenesis slide (#81506, IBD) and incubated at 37°C for 30 min to allow the Matrigel to polymerize. The treated HUVECs were seeded into the Matrigel-coated well. The plate was then incubated at 37°C in 5% CO_2_ humidified atmosphere. Tube formation was observed at 12 h with microscope. The tube formation ability was determined by measuring the number of tubes. Each experiment was repeated three times.

### 2.7. Immunohistochemistry (IHC)

4 *μ*m thick sections from paraffin-embedded specimens were deparaffinized with xylenes and rehydrated. Then, sections were incubated with 3% H_2_O_2_ to quench the endogenous peroxidase activity and incubated with polyclonal antibodies against DNAJA1 (1 : 200, ab192904, Abcam, Cambridge, UK), Ki67 (1 : 1000, Cat No. 27309-1-AP, Proteintech), and CD31 (1 : 200, Cat No. 66065-2-Ig) overnight at 4°C, respectively. After incubation with secondary antibody at room temperature, the sections were stained with v 3,3′-diaminobenzidine tetrachloride (DAB) and hematoxylin. Two pathologists blinded to the clinical parameters reviewed and scored these sections. Intensity score (0: no staining, 1: weakly stained, 2: moderately stained, and 3: strongly stained) was multiplied by the density score (0: 0%, 1: 1–25%, 2: 26–50%, 3: 51–75%, and 4: >75%) to calculate an overall score (0-7). The staining of DNAJA1 was assessed as follows: (−) means a final staining score < 1; (+) a final staining score of 1~3; (+ +) a final staining score of 4~5; and (+ + +) a final staining score of 6~7. An optimal cut-off value was defined: the final staining score 0~+ was classified as low expression, and the final staining score ++~+++ was classified as high expression.

### 2.8. RNA Extraction and Real-Time RT-PCR

Total RNAs were isolated from cells or fresh tissues with TRIzol reagent (Invitrogen, Carlsbad, CA, USA) and reverse-transcribed into cDNA using iScript cDNA Synthesis Kits (#1708840) and TaqMan™ MicroRNA reverse transcription kit (#4366596). The PCR primers for DNAJA1 were as follows: forward: 5′-ATG TGC GGA ATC TTT GCC TAC-3′ and reverse: 5′-ATC GAG AGC CTT GAC TTT CC C-3′. The PCR primers for miR-205-5p were as follows: forward 5′-GCGGCGGTGTA GTGTTTC CTA-3′ and reverse 5′-GTGCAGGGTCCGAGGT-3′. RT-PCR was carried out using SYBR Premix Ex Taq II (TaKaRa, Japan) and measured in ABI 7500 Real-Time PCR System (Applied Biosystems, USA). GAPDH and U6 were used as an internal reference. Relative expression levels were calculated using 2-*ΔΔ*Ct method. All the reactions were run in triplicate.

### 2.9. Cell Cycle Analysis

1 × 10^6^ treated cells were collected, washed twice with PBS, and fixed in 1% ice-cold paraformaldehyde for 1 h. The samples were then centrifuged to remove the supernatant and stained with the solution contained with 100 *μ*g/ml RNaseA (Sigma, USA) and propidium iodide (PI) (Sigma, USA) for 30 min at 37°C. Cell cycle distributions were determined using flow cytometry.

### 2.10. In Vitro Apoptosis Assay

Treated cells were harvested and stained with Annexin V-FITC and propidium iodide (PI) according to the manufacturer's protocol (BioVision #K101–100). Annexin V-FITC/PI binding was analyzed by flow cytometry using BD FACS Calibur system, and data was analyzed using Cell Quest software.

### 2.11. Western Blotting

The cells were lysed on ice in RIPA buffer with protease inhibitor and phosphatase inhibitor followed by centrifugation at 12000 rpm, 4°C for 20 min to collect cell lysis. Total protein concentration was quantified by BCA. Cell lysates were loaded onto SDS/PAGE, transferred onto PVDF membranes, and incubated with anti-DNAJA1 polyclonal antibody (1 : 500, ab192904, Abcam, Cambridge, UK) and anti-EF1A1(1 : 500, ab212173, Cambridge, UK) antibody overnight at 4°C, followed by incubation with secondary antibodies. A mouse monoclonal anti-GAPDH (1 : 1000) (Sigma-Aldrich, St. Louis, USA) was used as an internal control.

### 2.12. Co-Immunoprecipitation (Co-IP)

Cell lysis was incubated 2 h at 4°C with IgG and protein A+G agarose to minimize unspecific binding. DNAJA1 (1 : 300, 1 : 500, ab192904, Abcam, Cambridge, UK) and EF1A1 (1 : 500, ab212173, Cambridge, UK) antibodies were then added at 4°C overnight. The protein A/G agarose was collected by centrifugation. Immunoprecipitated proteins were analyzed by SDS-PAGE (10%, Minigel) at 100 V for 1.5 h. DNAJA1 and EF1A1 antibodies were diluted, respectively, and incubated with membranes at 4°C overnight. The secondary antibodies were then incubated for 1 h at room temperature. Protein bands were visualized using enhanced chemiluminescence (PerkinElmer Life Sciences). The experiments were repeated three times.

### 2.13. Luciferase Activity Assay

DNAJA1 3′UTR segments were amplified by PCR and inserted into the pGL3-basic vector. Mutant constructs in miR-205-5p binding sites of DNAJA1 3′UTR region were generated using Quick Change Site-Directed Mutagenesis Kit (Agilent, Roseville City, CA). Cotransfections of miR-205-5p lentivirus vector with wild-type DNAJA1 3′UTR or mutant DNAJA1 3′UTR plasmid were performed by using Lipofectamine 3000 (Invitrogen). Luciferase activity was measured 48 h after transfection using the Dual-Luciferase Reporter Assay System (Promega). Each assay was repeated in 6 independent experiments.

### 2.14. Statistical Analysis

The expression of DNAJA1 in liver tissues was analyzed with paired samples *t* test. The correlation of DNAJA1 expression with various clinicopathological parameters was evaluated with chi-square test. Survival analyses were performed according to the Kaplan-Meier method and compared using the log-rank test. Cell proliferation and in vitro invasion assays were analyzed using one-way ANOVA. SPSS 23.0 software was used for all statistical analyses and *P* < 0.05 was considered significant.

## 3. Results

### 3.1. DNAJA1 Promotes the Proliferation of Liver Cancer Cells In Vitro

**Figure 1:**
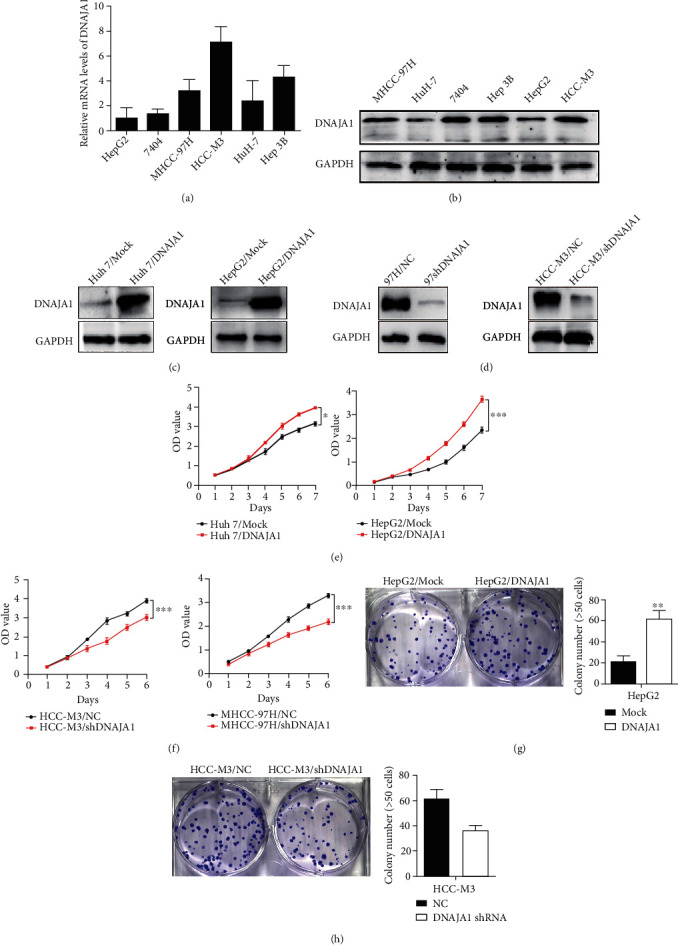
DNAJA1 promoted proliferation of liver cancer cells in vitro. (a) The mRNA level of DNAJA1 in six liver cancer cell lines detected by q-PCR. (b) The protein expression of DNAJA1 in six liver cancer cell lines detected by western blot. Error bars represent mean ± SD from three independent experiments. ^∗^*P* < 0.05. (c) Relative protein expressions of DNAJA1 in Huh 7 and HepG2 cells stably transfected with DNAJA1 overexpression vector or control vector by western blot. (d) Relative protein expressions of DNAJA1 in MHCC-97H and HCC-M3 cells stably transfected with DNAJA1 shRNA or negative control by western blot. (e and f) Effect of DNAJA1 overexpression or downregulation on cell proliferation of Huh 7 and HepG2 cells or MHCC-97H and HCC-M3 cells detected by CCK-8 assay. (g and h) Effect of DNAJA1 overexpression or inhibition on cell clone formation ability of HepG2 cells or HCC-M3 cells.

**Figure 2:**
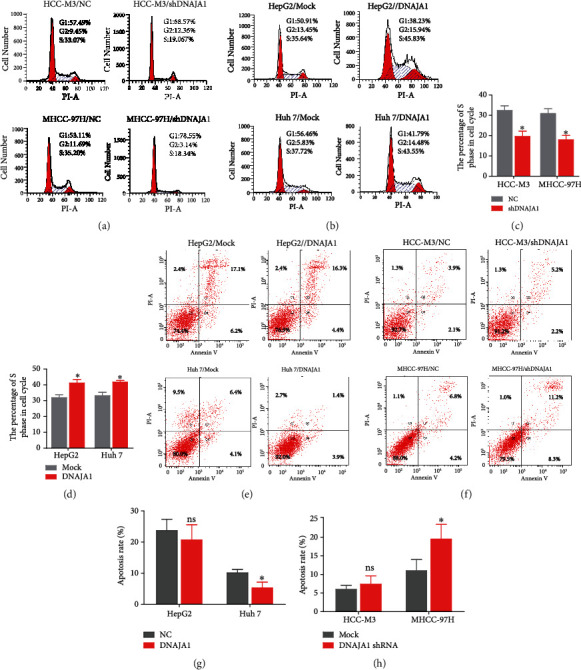
DNAJA1 promotes the proliferation by arresting the tumor cells at the S and G2/M phase and reducing apoptosis. (a) Representative histograms depicting cell cycle profiles of MHCC-97H and HCC-M3 cells stably transfected with DNAJA1 shRNA or negative control. (b) Representative histograms depicting cell cycle profiles of Huh 7 and HepG2 cells stably transfected with DNAJA1 overexpression vector or control vector. (c and d) The quantification of the effect of DNAJA1 on cell cycle. (e) Percent of apoptosis in Huh 7 and HepG2 cells stably transfected with DNAJA1 overexpression vector or control vector. (f) Percent of apoptosis in MHCC-97H and HCC-M3 cells stably transfected with DNAJA1 shRNA or negative control. (g and h) The quantification of the effect of DNAJA1 on apoptosis. Error bars represent mean ± SD from three independent experiments. ^∗^*P* < 0.05.

To investigate the role of DNAJA1 in liver cancer progression, we first detected endogenous expression of DNAJA1 in six liver cancer cell lines. DNAJA1 was highly expressed in HCC-M3 and MHCC-97H cell lines with high metastatic potential and expressed at lower levels in Hun 7 and HepG2 cells with weak metastatic potential (Figures 1(a) and 1(b)). Gain-of-function experiments were performed using exogenous expression plasmids in Huh 7 and HepG2 cells, and loss-of-function was conducted using shRNA in 97H and HCC-M3 cells (Figures 1(c) and 1(d)). CCK-8 assays exhibited that DNAJA1 overexpression increased the cell proliferation as depicted by corresponding OD values in Huh 7 and HepG2 cells (Figure 1(e)). Conversely, knocking down of DNAJA1 in MHCC-97H and HCC-M3 cells showed decreased cell proliferation (Figure 1(f)). Colony formation assays further confirmed the effect of DNAJA1 on promoting proliferation rate (Figures 1(g)–1(h)). Furthermore, depletion of DNAJA1 significantly increased the proportion of cells at the G1/G0 phase and reduced the proportion of cells at the S and G2/M phase (Figures 2(a) and 2(c), *P* < 0.05), whereas Huh 7 and HepG2 cells with forced DNAJA1 expression showed reverse effects (Figures 2(b) and 2(d), *P* < 0.05). These data suggested that DNAJA1 promotes liver cancer cell proliferation by provoking S and G2/M peak. The percentage of apoptosis was significantly decreased in DNAJA1-overexpressed Huh cells (*P* < 0.05, Figures 2(e) and 2(g)), while depletion of DNAJA1 in MHCC-97H cells exhibited more apoptotic cells (Figures 2(f) and 2(h)). All the results above indicate that DNAJA1 promotes cell proliferation by regulation of the cell cycle and inhibiting apoptosis.

### 3.2. DNAJA1 Enhances the Invasion, Metastasis, and Angiogenesis of Liver Cancer Cells

**Figure 3:**
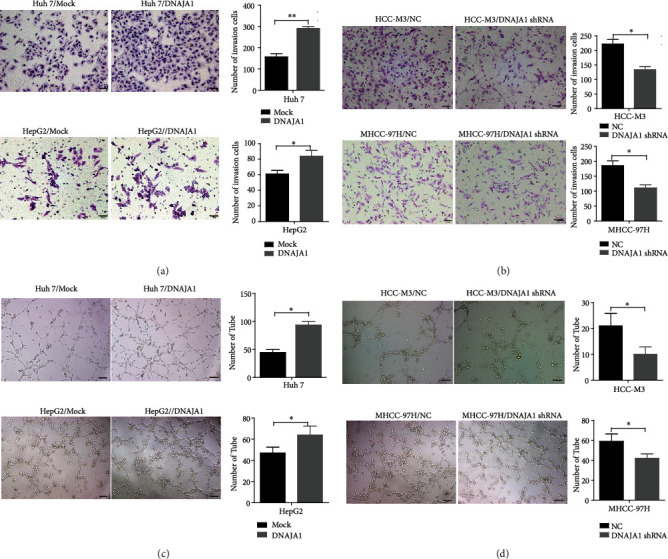
DNAJA1 promoted invasion and angiogenesis of liver cancer cells in vitro. (a) The effect of DNAJA1 on invasion in Huh 7 and HepG2 cells stably transfected with DNAJA1 overexpression vector or control vector. (b) The effect of DNAJA1 on invasion in MHCC-97H and HCC-M3 cells stably transfected with DNAJA1 shRNA or negative control. (c) The effect of DNAJA1 on angiogenesis in Huh 7 and HepG2 cells stably transfected with DNAJA1 overexpression vector or control vector. (d) The effect of DNAJA1 on angiogenesis in MHCC-97H and HCC-M3 cells stably transfected with DNAJA1 shRNA or negative control.

Next, we assessed the role of DNAJA1 in the invasion and angiogenesis of liver cancer. We found that upregulation of DNAJA1 showed a markedly increase of invasion in Huh 7 and HepG2 cells, while downregulation of DNAJA1 showed opposite effects (Figures 3(a) and 3(b), *P* < 0.05). In addition, DNAJA1 overexpression enhanced tube formation in the human umbilical vein endothelial cells (HUVEC) compared to the control group (Figure 3(c), *P* < 0.05), which were revered by depletion of DNAJA1 (Figure 3(d), *P* < 0.05). These results together indicate that DNAJA1 promotes the invasion and angiogenesis of liver cancer cells.

**Figure 4:**
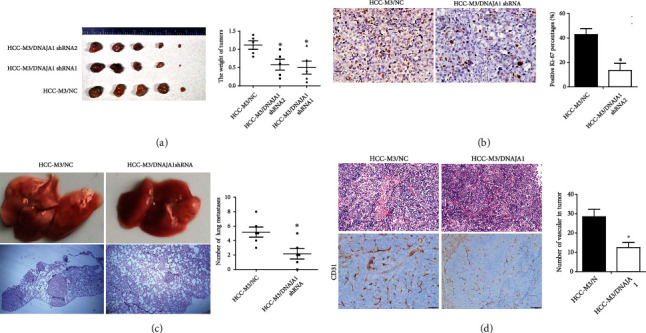
DNAJA1 promoted proliferation and metastasis of liver cancer cells in vivo. (a) Images and statistical analysis of subcutaneous tumor formed by HCC-M3 cells stably transfected with DNAJA1 shRNA or negative control. Qualification of tumor weight in HCC-M3/DNAJA1 shRNA or HCC-M3/NC groups. (b) Representative images of Ki67 staining in subcutaneous tumor sections by immunohistochemistry. (c) The effect of DNAJA1 overexpression on metastasis in HCC-M3/DNAJA1 shRNA or HCC-M3/NC groups. (d) The effect of DNAJA1 overexpression on angiogenesis in HCC-M3/DNAJA1 shRNA or HCC-M3/NC groups. Error bars represent mean ± SD from three independent experiments. ^∗^*P* < 0.05.

We then evaluated the effect of DNAJA1 on tumor growth, metastasis, and angiogenesis in vivo. HCC-M3 cells with or without DNAJA1-shRNA expression were injected subcutaneously into nude mice, respectively, and then, the resultant tumors were collected and weighed. It is shown that tumors in mice injected with DNAJA1 shRNA-expressing cells grew more slowly than in the control group (Figure 4(a)). IHC staining displayed much lower positive Ki-67 percentages in tumors with DNAJA1 depletion than those in the control group (Figure 4(b)). To examine the effect of DNAJA1 on liver cancer metastasis in vivo, the cells were transplanted into the liver of nude mice, and the number of lung metastases in HCC-M3/DNAJA1 shRNA group was obviously smaller than the control group (Figure 4(c), *P* < 0.05). Moreover, there were less number of vascular in primary tumor of liver in DNAJA1 shRNA group than that in the control group (Figure 4(d)*P* < 0.05). In summary, DNAJA1 profoundly boosts tumor growth and metastasis in vivo.

### 3.3. DNAJA1 Was a Direct Target of miR-205-5p and It Stabilized EF1A1

**Figure 5:**
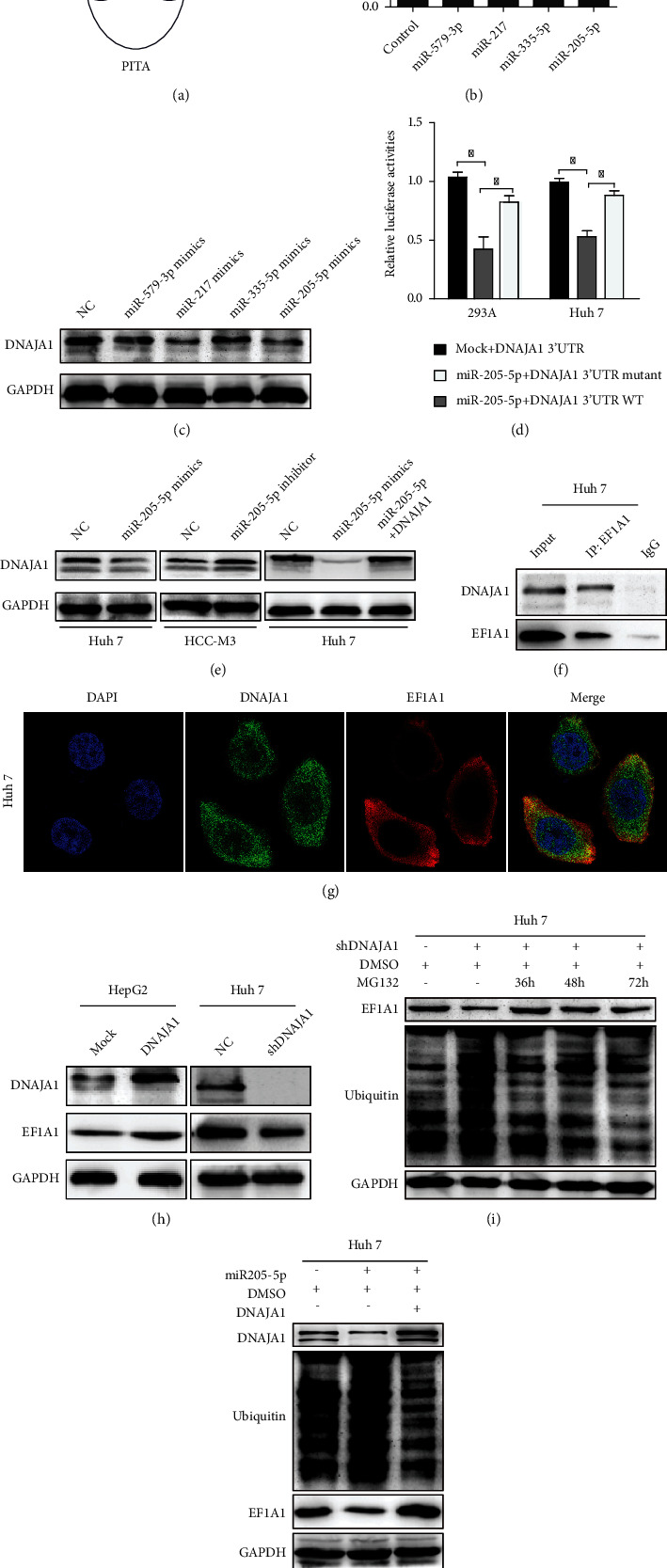
DNAJA1 was directly targeted by miR-205-5p and interacted with EF1A1. (a) Predicted microRNAs which targeted to DNAJA1 were predicted by three common bioinformatic algorithms. (b) Luciferase assay analyses of the indicated cells transfected with the indicated reporters with miR-205-5p, miR335-5p, miR579-3p, and miR-217. (c) Western blot analysis of DNAJA1 in HCC-M3 cells transfected with miR-205-5p, miR335-5p, miR579-3p, and miR-217 mimics. (d) Luciferase assay analyses of HCC-M3 cells simultaneously transfected with the 3′-UTRs of WT and mutant DNAJA1 and miR-205-5p mimics. (e) Western blot analysis of DNAJA1 in HCC-M3 cells transfected with miR-205-5p with or without DNAJA1. (f) Mutual combination between DNAJA1 and EF1A1 in Huh 7 cells by Co-IP assay. (g) Colocalization of DNAJA1 and EF1A1 by immunofluorescence (blue: DAPI, green: DNAJA1, and red: EF1A1). (h) The protein level of DNAJA1 and EF1A1 in DNAJA1 overexpressing or inhibiting cells by western blot. (i) Effect of time-course inhibition of DNAJA1 on the expression of EF1A1 and the status of its ubiquitination by western blot. (j) Effect of miR-205-5p on the expression of EF1A1 and DNAJA1 and ubiquitin in HCC-M3 cells by western blot. GAPDH was used as internal control.

Posttranscriptional gene regulation is often mediated by microRNAs. Using three common bioinformatic algorithms (TargetScan, Pictar, and miRcode), we attempted to forecast the miRNAs that may directly target DNAJA1 at 3′UTR. About >20 miRNAs were screened out to bind to 3′UTR of DNAJA1. Among those miRNAs, miR-205-5p, miR335-5p, miR579-3p, and miR-217 were predicted by all three databases (Figure 5(a)). Therefore, we cotransfected mimics of these miRNAs with the wild-type (WT) 3′UTR of DNAJA1 into Huh 7 cells. Reporter assays confirmed that miR-205-5p markedly reduced the luciferase activity of DNAJA1 3′UTR (Figure 5(b)) and the protein level of DNAJA1 (Figure 5(c)), which were not affected by mutant 3′UTR of DNAJA1 (Figure 5(d)). In addition, ectopic miR-205-5p in Huh 7 cells downregulated DNAJA1 expression, whereas overexpression of DNAJA1 rescued this effect, while miR-205-5p inhibitor increased expressions of DNAJA1 (Figure 5(e)). Hence, miR-205-5p could directly target DNAJA1and was selected for the following study.

In searching interacted targets of DNAJA1by immunoprecipitation and mass spectrometer data, we found that eukaryotic translation elongation factor 1 alpha 1 (EF1A1) showed highest score in peptide fragment blast (Figure S1A and S1B, Table S1). Co-IP assays validated that DNAJA1 could directly bind with EF1A1 (Figure 5(f)), and the complex was colocalized in the cytoplasm in HCC-M3 cells (Figure 5(g)). Moreover, overexpression and depletion of DNAJA1 increased and decreased the protein level of EF1A1 in HepG2 and HCC-M3 cells, respectively (Figure 5(h)). Thus, we speculated whether DNAJA1 regulates the stabilization and ubiquitin degradation of EF1A1. The results showed that knockdown of DNAJA1 led to increased recovery of ubiquitinated EF1A1, and MG132 treatment reduced the ubiquitination of EF1A1 (Figure 5(i)). Consistently, miR-205-5p also exacerbated the ubiquitin of EF1A1 and overexpression of DNAJA1 reversed this effect (Figure 5(j)). Taken together, these results demonstrate that DNAJA1 mediates the ubiquitination of EF1A1 via protein interaction in liver cancer.

**Figure 6:**
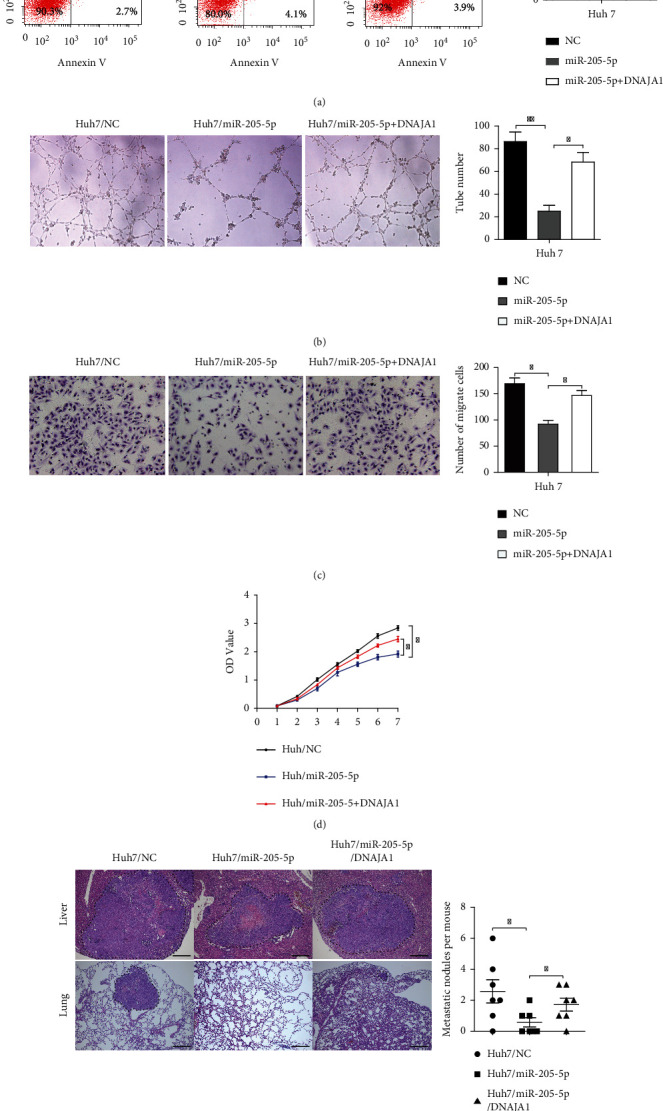
DNAJA1 reversed the effect of miR-205-5p on the liver cells. (a) Representative histograms depicting cell apoptosis of HCC-M3 cells stably transfected with miR-205-5p with or without DNAJA1 shRNA. (b) Tubule formation assays of HCC-M3 cells stably transfected with miR-205-5p with or without DNAJA1 shRNA. (c) Transwell assays of HCC-M3 cells stably transfected with miR-205-5p with or without DNAJA1 shRNA. (d) CCK8 assays of HCC-M3 cells stably transfected with miR-205-5p with or without DNAJA1 shRNA. (e) Images and statistical analysis of liver subcapsular tumor implantation of Huh 7 cells stably transfected with miR-205-5p or miR-205-5p/DNAJA1 shRNA or negative control.

Additionally, we wondered whether miR-205-5p regulates the function of DNAJA1 and showed that all suppressive effects of miR-205-5p on cell cycle, angiogenesis, invasion, and proliferation could be reversed by DNAJA1 overexpression (Figures 6(a)–6(d), *P* < 0.05). In vivo assays also verified that miR-205 could indeed regulate DNAJA1-induced proliferation and metastasis of liver cancer cells (Figure 6(e)). The above data uncover that miR-205-5p transcriptionally regulates DNAJA1 to stabilize EF1A1.

### 3.4. DNAJA1 Is Upregulated in Liver Cancer and Predicts Poor Prognosis for Liver Cancer Patients

**Figure 7:**
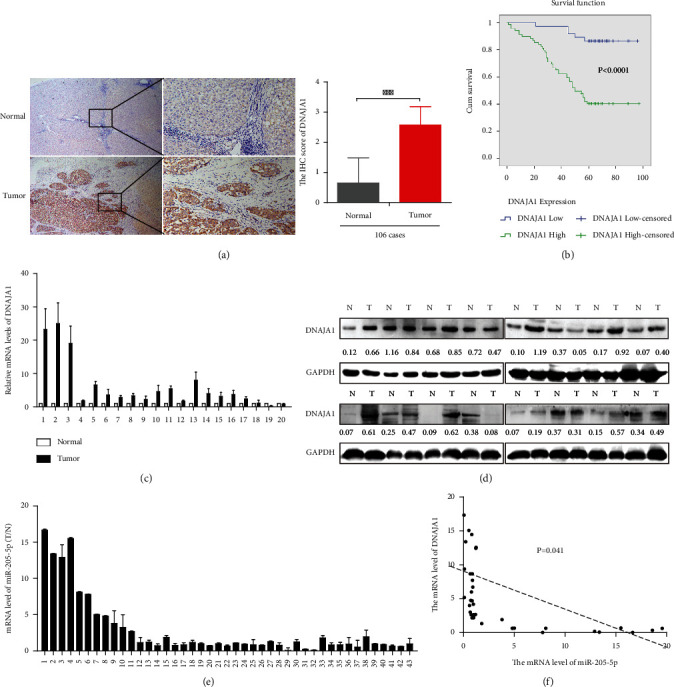
DNAJA1 is upregulated in liver cancer and negatively related to miR-205-5p. (a) Representative images of DNAJA1 proteins in 106 cases of paraffin-embedded liver cancer specimens by immunohistochemistry. (b) High expression of DNAJA1 associated with shorter survival of liver cancer patients by the Kaplan-Meier analysis. (c and d) DNAJA1 mRNA and protein level in 20 cases (presented) of fresh liver cancer tissues and corresponding normal liver detected by qPCR and western blotting. (e) Detection of miR-205-5p level in 43 cases of paired fresh HCC patients by real-time PCR. (f) The relationship between miR-205-5p and DNAJA1. Error bars represent mean ± SD from three independent experiments.

We explored the expression pattern of DNAJA1 and its clinicopathologic value in liver cancer. DNAJA1 expression was first examined by IHC in 106 cases of paraffin-embedded liver cancer tissues. Strong staining for DNAJA1 was frequently observed in liver cancer (Figure 7(a)). Statistically, the expression of DNAJA1 was much stronger in liver cancer in contrasted with normal liver (Figure 7(a), *P* < 0.0001) and was significantly higher in cancer tissues than those in adjacent normal or cirrhotic livers (*t* = −8.233, *P* < 0.001; *t* = −14.95, *P* < 0.001, respectively, Table S2), although no significant difference was observed between cancer tissues with and without cirrhosis (*t* = 0.820, *P* = 0.414, Table S2). The expression of DNAJA1 was associated with clinicopathologic features in liver cancer (Table 1) and correlated strongly with differentiation (*P* < 0.001), dissemination (*P* < 0.001), and serum AFP (*P* = 0.020). The Kaplan Meier survival analysis showed that higher expression of DNAJA1 predicted poorer survival for liver cancer patients (Figure 7(b), *P* < 0.001). In addition, both the univariate and multivariate Cox proportional hazard model indicated that high expression of DNAJA1, portal vein thrombosis, and intrahepatic dissemination were predictive factors for HCC (Table S3, S4). DNAJA1 was obviously upregulated in 43 cases of fresh liver cancer tissues compared to normal livers (*P* = 0.003) and was either unaltered or downregulated in three cases of cancer tissues versus normal (Figure 7(c)). Higher DNAJA1 expression in liver cancer tissues compared to normal was also validated by western blots (Figure 7(d)). The expression levels of miR-205-5p were downregulated in 43 cases of paired fresh liver cancer tissues (Figure 7(e)) and were negatively correlated to the mRNA levels of DNAJA1 (Figure 7(f)). In summary, the upregulation of DNAJA1 correlated with liver cancer progression and could be an independent prognostic indicator for survival of liver cancer patients.

## 4. Discussion

In this study, we explored the functional role of DNAJA1 and the mechanism of regulation in liver cancer. DNAJA1 is a member of DnaJ protein family and interacts with HSP70 to regulate ATP hydrolysis activity via its J domain. It is responsible for many cell activities, such as unfolding misfolded protein, polypeptide chain folding, transmembrane transport of polypeptide, assembling and disassembling protein complex, and regulation of protein formation [13, 14]. In recent years, emerging in tumor-related fields, DNAJA1 has been identified as biomarker for pancreatic cancer and breast cancer [12, 15]. DNAJA1 promotes cancer metastasis through interaction with mutant p53 in head and neck squamous cell carcinoma [16] and enhances the radiotherapy resistance of glioma cells [17]. Our work firstly demonstrates that DNAJA1 promotes liver cancer progression. It was highly expressed in liver cancer and closely related with differentiation, dissemination, and serum AFP. As for tumorigenesis, DNAJA1 promoted the proliferation, invasion, and angiogenesis of liver cancer cells in vitro and vivo, possibly by regulation of cell cycle and apoptosis of HCC cells, although the influences of DNAJA1 on apoptosis of these liver cancer cells vary. Knockdown or overexpression of DNAJA1 showed drastic influence on the apoptosis in MHCC-97H and Huh 7 cell lines, but not in in HepG2 and HCC-M3 cell lines, possibly due to distinct origins of these cell lines.

Next, we clarified the potential mechanisms of actin of DNAJA1 in liver cancer. Increasingly, studies have elucidated that microRNAs (miRNAs), a class of small-regulatory RNA molecules, posttranscriptionally regulate the expression of target mRNA by binding to the 3′-untranslated region (UTR). For instance, miRNA-21, miRNA-378, and miRNA-99b have been shown to contribute to hepatic cancer progression [18–20]. Here, using database predication and functional validation, we discovered that DNAJA1 was directly mediated by miR-205-5p. Previously, miR-205-5p has been reported to suppress progression of tumor such as colorectal cancer, breast cancer, and prostatic cancer [21–23]. In this study, we found that miR-205-5p was downregulated in liver cancer, whereas DNAJA1 was highly expressed in liver cancer and negatively correlated with the expression of miR-205-5p, confirming that DNAJA1 was a target gene of miR-205-5p.

Meanwhile, we performed immunoprecipitation and mass spectrometer assays and revealed that DNAJA1 could bind with EF1A1. EF1A1 participates in extension of the peptide chain in the cell. And it is also involved in cell apoptosis [24, 25] and migration of tumor cells [26]. Previous studies reported that EF1A1 could interact with HSC70 to cooperatively suppress brain endothelial cell apoptosis [25]. Our data further proved that DNAJA1 stabilizes EF1A1 protein to prevent its ubiquitination-mediated degradation. Based on the above results, we hypothesized that DNAJA1 promotes the proliferation and metastasis of liver cancer by preventing the ubiquitination and degradation of EF1A1.

In conclusion, our study demonstrates that upregulation of DNAJA1 in liver cancer associates with poor survival of patients. As a target of miR-205-5p, DNAJA1 acts as an oncogene in the progression of liver cancer via binding to EF1A1 to reduce the ubiquitination and degradation of EF1A1. Therefore, DNAJA1 may be a significant (and promising?) biomarker for liver cancer progression.

## Figures and Tables

**Table 1:** Relationship between DNAJA1 expressions and clinicopathologic features of hepatocellular carcinoma patients.

Features	Total number	High expression	Low expression	*P*	*λ* ^2^
All case	106	69	37		
Age				0.187	1.793
<55		48	21		
≥55		21	16		
Gender				0.052	3.778
Male		62	28		
Female		7	9		
Tumor size				0.148	2.096
<5 cm		29	21		
≥5 cm		40	16		
Differentiation				<0.001	34.025
Well		5	21		
Moderate		47	15		
Poor		17	1		
Cirrhosis				0.853	0.034
N		36	20		
Y		33	18		
Distant metastasis				0.591	0.289
N		43	26		
Y		26	12		
Relapse				0.324	0.974
N		36	23		
Y		23	14		
Portal vein thrombosis				0.075	3.174
N		54	34		
Y		15	3		
Intrahepatic dissemination				<0.001	13.843
N		28	29		
Y		41	8		
HBsAg				0.896	0.017
Negative		16	9		
Positive		53	28		
Serum AFP				0.029	4.76
>25 ng		45	16		
≦25 ng		24	21		

## Data Availability

All data generated or analyzed during this study are included in this published article and its supplementary information files.

## Supplementary Materials

Supplementary Materials Other experimental methods and data are shown in Supplemental materials.
