# Supplementary material for: DNAJA1 Stabilizes EF1A1 to Promote Cell Proliferation and Metastasis of Liver Cancer Mediated by miR-205-5p
Source: J Oncol. 2022 May 9;2022:2292481. doi: 10.1155/2022/2292481 (PMC9110222; doi:10.1155/2022/2292481)
Supplement: Supplementary Materials — Other experimental methods and data are shown in Supplemental materials. [file 2292481.f1.zip › Supplementary file (2).docx]

**Supplementary figures and figure legends**


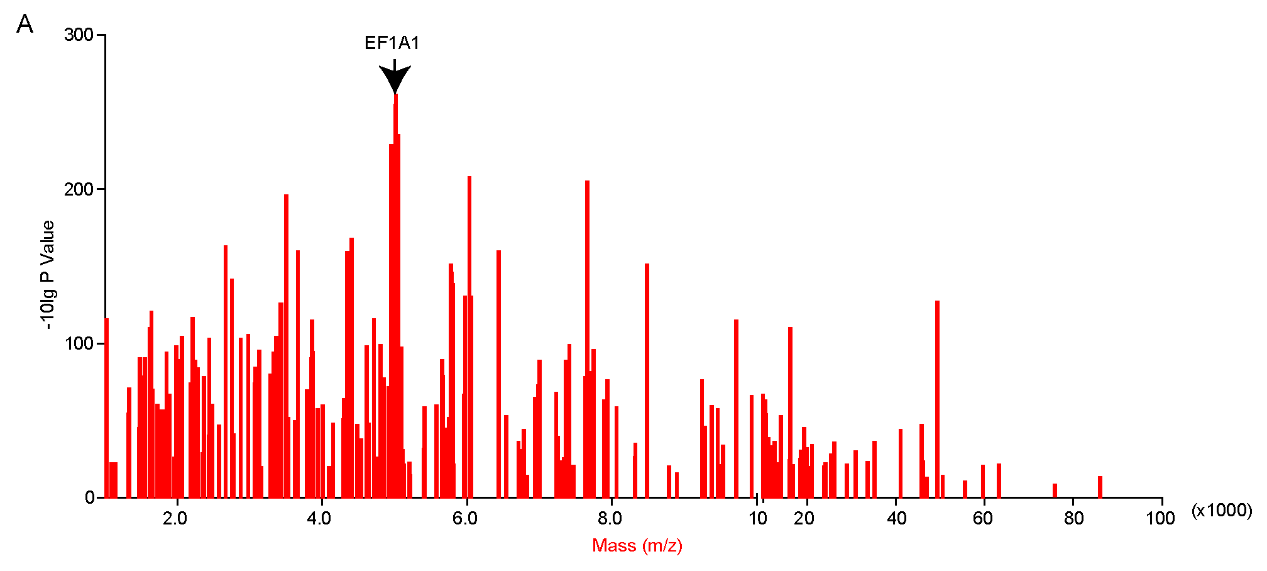


**Figure S1. The expression of DNAJA1 in liver cancer cells.** (A) Potential genes interact with DNAJA1 by immunoprecipitation followed by mass spectrometry.
