# Supplementary material for: DNAJA1 Stabilizes EF1A1 to Promote Cell Proliferation and Metastasis of Liver Cancer Mediated by miR-205-5p
Source: J Oncol. 2022 May 9;2022:2292481. doi: 10.1155/2022/2292481 (PMC9110222; doi:10.1155/2022/2292481)
Supplement: Supplementary Materials — Other experimental methods and data are shown in Supplemental materials. [file 2292481.f1.zip › Supplementary Tables.docx]

**Supplementary tables**

**Table S1. Top 30 of the possible genes interacted with DNAJA1 by mass spectrometry.**

| Accession | -10lgP | Avg. Mass |
| --- | --- | --- |
| EF1A1_HUMAN | 267.24 | 5020.38 |
| EF1A3_HUMAN | 264.67 | 5010.3 |
| ACTG_HUMAN | 253.36 | 41793 |
| TBB4B_HUMAN | 252.84 | 49831 |
| TBB3_HUMAN | 224.47 | 50433 |
| ENOA_HUMAN | 203.07 | 7847.5 |
| KV401_HUMAN | 199.68 | 3776.4 |
| ADT2_HUMAN | 189.12 | 4304.8 |
| CYTS_HUMAN | 176.62 | 2637.5 |
| CYTN_HUMAN | 164.27 | 6361.5 |
| ENOA_HUMAN | 162.5 | 3878.5 |
| CDC45_HUMAN | 161.25 | 4239.5 |
| EF1G_HUMAN | 157.34 | 5813.5 |
| PTRF_HUMAN | 157.09 | 8368.5 |
| LDHA_HUMAN | 155.14 | 5802.3 |
| EF2_HUMAN | 149.91 | 2434.5 |
| K22E_HUMAN | 147.35 | 5800.4 |
| ARF3_HUMAN | 130.89 | 5964.5 |
| ARF1_HUMAN | 129.88 | 6060.5 |
| RS15A_HUMAN | 125.92 | 3648.2 |
| TCPZ_HUMAN | 125.58 | 9802.4 |

**Table S2. The expression of DNAJA1 in liver cancer samples by IHC.**

| Group | DNAJA1 expression | | | | Total |
| --- | --- | --- | --- | --- | --- |
|  | - | + | ++ | +++ |  |
| None -tumor | 53 | 28 | 21 | 4 | 106 |
| Cirrhosis tissue  Normal tissue | 12  41 | 12  16 | 6  15 | 4  0 | 34  72 |
| tumor | 0 | 7 | 33 | 66 | 106 |
| Liver tumor with cirrhosis  Liver Tumor without cirrhosis | 0  0 | 1  6 | 12  21 | 21  45 | 34  72 |

Normal tissue VS tumor t=-14.995, P<0.001; （paired samples T test）

Cirrhosis tissue VS Tumor t=-8.053, P<0.001 （paired samples T test）

Tumor with cirrhosis VS tumor without cirrhosis t=0.820, P=0.414 （Independent sample t test）

**Table S3 Univariate analyses of individual parameters for associations with overall survival rate: Cox proportional hazards model**

| Variables | Univariate | | P value |
| --- | --- | --- | --- |
|  | HR | CI(95%) |  |
| DNAJA1 (high vs low) | 6.305 | 2.486-15.991 | 0.000 |
| Age (≥55 vs. <55) | 0.870 | 0.469-1.611 | 0.6570 |
| Gender (male vs. female) | 0.35 | 0.100-1.129 | 0.079 |
| Differentiation (poor vs. moderate vs. well) | 1.636 | 1.082-2.474 | 0.020 |
| Distant metastasis (Yes vs. No) | 2.121 | 1.188-3.789 | 0.011 |
| Tumor size (>=5cm vs. <5cm) | 1.793 | 0.985-3.265 | 0.056 |
| Cirrhosis (None vs. Yes) | 0.877 | 0.468-1.643 | 0.682 |
| Relapse (Yes vs. No) | 2.920 | 1.588-5.371 | 0.001 |
| Portal Vein Thrombosis  (Yes vs. No) | 4.329 | 2.309-8.115 | 0.000 |
| Intrahepatic Dissemination  (Yes vs. No) | 3.335 | 1.792-6.206 | 0.000 |
| HBsAg (Positive vs. Negative) | 0.778 | 0.403-1.503 | 0.445 |
| Serum AFP (>25ng vs. ≦25ng) | 1.546 | 0.842-2.836 | 0.160 |

**Abbreviations:**

HR, hazard ratio; CI, confidence interval.

**Table S4 Multivariate analyses of individual parameters for associations with overall survival rate: Cox proportional hazards model**

| Variables | Multivariate | | P value |
| --- | --- | --- | --- |
|  | HR | CI(95%) |  |
| DNAJA1(high vs low) | 5.198 | 2.003-13.491 | 0.001 |
| intrahepatic dissemination (Yes vs. No) | 2.062 | 1.072-3.968 | 0.030 |
| Portal Vein Thrombosis  (Yes vs. No) | 2.605 | 1.381-4.916 | 0.003 |
